# Directional seed and pollen dispersal and their separate effects on anisotropy of fine‐scale spatial genetic structure among seedlings in a dioecious, wind‐pollinated, and wind‐dispersed tree species, *Cercidiphyllum japonicum*

**DOI:** 10.1002/ece3.7609

**Published:** 2021-05-07

**Authors:** Atsushi Nakanishi, Susumu Goto, Chikako Sumiyoshi, Yuji Isagi

**Affiliations:** ^1^ Hokkaido Research Center, Forestry and Forest Products Research Institute Forest Research and Management Organization Sapporo Japan; ^2^ Education and Research Center The University of Tokyo Forests Graduate School of Agricultural and Life Sciences The University of Tokyo Tokyo Japan; ^3^ Faculty of Integrated Arts and Sciences Hiroshima University Higashi‐Hiroshima Japan; ^4^ Present address: DeepL GmbH Cologne Germany; ^5^ Present address: Graduate School of Agriculture Kyoto University Kyoto Japan

**Keywords:** anemochory, anemophily, gene flow, microsatellite, neighborhood model

## Abstract

Prevailing directions of seed and pollen dispersal may induce anisotropy of the fine‐scale spatial genetic structure (FSGS), particularly in wind‐dispersed and wind‐pollinated species. To examine the separate effects of directional seed and pollen dispersal on FSGS, we conducted a population genetics study for a dioecious, wind‐pollinated, and wind‐dispersed tree species, *Cercidiphyllum japonicum* Sieb. et Zucc, based on genotypes at five microsatellite loci of 281 adults of a population distributed over a ca. 80 ha along a stream and 755 current‐year seedlings. A neighborhood model approach with exponential‐power‐von Mises functions indicated shorter seed dispersal (mean = 69.1 m) and much longer pollen dispersal (mean = 870.6 m), effects of dispersal directions on the frequencies of seed and pollen dispersal, and the directions with most frequent seed and pollen dispersal (prevailing directions). Furthermore, the distance of effective seed dispersal within the population was estimated to depend on the dispersal direction and be longest at the direction near the prevailing direction. Therefore, patterns of seed and pollen dispersal may be affected by effective wind directions during the period of respective dispersals. Isotropic FSGS and spatial sibling structure analyses indicated a significant FSGS among the seedlings generated by the limited seed dispersal, but anisotropic analysis for the seedlings indicated that the strength of the FSGS varied with directions between individuals and was weakest at a direction near the directions of the most frequent and longest seed dispersal but far from the prevailing direction of pollen dispersal. These results suggest that frequent and long‐distance seed dispersal around the prevailing direction weakens the FSGS around the prevailing direction. Therefore, spatially limited but directional seed dispersal would determine the existence and direction of FSGS among the seedlings.

## Graphical abstract

We conducted a population genetics study for a dioecious, wind‐pollinated, and wind‐dispersed tree species based on genotypes at five microsatellite loci of 281 adults of a population distributed over a ca. 80 ha along a stream and 755 current‐year seedlings. The results indicated directionalities of gene dispersal via seeds and pollen separately, and effect of the directional seed dispersal on anisotropy of a fine‐scale spatial genetic structure among the seedlings.

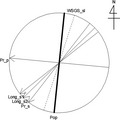

## 1 INTRODUCTION

Gene dispersal via seeds and pollen largely affect fine‐scale spatial genetic structure (FSGS) of plant populations, and the FSGS reflects Wright's neighborhood size (Wright, 1943, 1946); for example, the *S*p statistic expressing the intensity of the FSGS estimates the inverse of the neighborhood size under certain assumptions (Vekemans & Hardy, 2004). Therefore, studies on gene dispersal and the consequent effects on the FSGS are highly important to clarify the evolutionary dynamics of plant populations. Although the FSGS of plant populations is a consequence of various ecological factors (Hamrick et al., 1993; Hardy et al., 2006; Ueno et al., 2006; Vekemans & Hardy, 2004), seed dispersal critically affects the shaping of the FSGS (Browne et al., 2018; Grivet et al., 2009; Nakanishi et al., 2009), as it transports both male and female gametes to determine the final locations of genotypes (Browne et al., 2018; Grivet et al., 2009), and it is more spatially restricted compared to pollen dispersal in many species (e.g., Browne et al., 2018; Nakanishi et al., 2009). Pollen dispersal, in contrast, could affect the extent of FSGS, in that, for example, distance‐dependent pollination increases the proportion of full siblings in aggregates of maternal siblings around each seed parent (Nakanishi et al., 2009). Such separate effects of seed and pollen dispersal on the FSGS have not been fully elucidated (but see, e.g., Browne et al., 2018), whereas the combined effect of both seed and pollen dispersal, sometimes along with other ecological factors, has been examined in many studies (Born et al., 2012; Hamrick et al., 1993; Hardy et al., 2006; Ueno et al., 2006; Vekemans & Hardy, 2004). Understanding such separate effects requires the elucidation of seed and pollen dispersal patterns and the consequent FSGS of dispersed and established individuals originated from the estimated gene‐dispersals, simultaneously.

Much knowledge on distances of gene dispersal via seeds and pollen has been gained using highly polymorphic molecular markers (Hardy, 2009). However, only a limited number of studies have examined the direction of gene dispersal via pollen (Austerlitz et al., 2007; Burczyk et al., 1996, 2004; Burczyk & Prat, 1997), and the direction of gene dispersal via seeds has rarely been studied (but see García et al., 2007). In addition, the relationship between the distance and direction of effective gene dispersal has not been examined, to our knowledge, likely because the basic model (e.g., Tufto et al., 1997) assumed that the probability of dispersal direction is independent of dispersal distance. This kind of relationship would be important for the genetic dynamics of populations, because genetic isolation by distance would be reinforced or prevented in the direction of short‐ or long‐distance gene dispersal, respectively (Born et al., 2012). Further, most studies of the FSGS, typically expressed as the relationship between kinship and the physical distance between individuals (Vekemans & Hardy, 2004), have been conducted by isotropic spatial autocorrelation analyses (Born et al., 2012). Such analyses cannot elucidate the effects of the directionality of gene dispersal on the FSGS. In wind‐dispersed and/or wind‐pollinated species, wind direction affects the frequencies of seed dispersal (Bullock & Clarke, 2000) and/or pollen dispersal (Damialis et al., 2005; Silva Palacios et al., 2000) in particular directions. Further, Born et al. (2012) expected in their study on the anisotropy of the FSGS that the strength of FSGS varied depending on directions and was weakest and strongest at the directions aligned with and orthogonal to the prevailing wind direction, respectively, in the wind‐pollinated and wind‐dispersed species. For such species, long‐distance gene dispersal would occur at the effective wind direction, that is, the distance of gene dispersal would depend on the dispersal direction, and consequently, the FSGS in that direction is considered weak due to overlapping gene shadows (Born et al., 2012). However, such separate effects of directionalities of seed or pollen dispersal on FSGS have not been elucidated.

For populations distributed relatively long but narrow, often seen in the riparian forest along a stream, the directions of potential seed and pollen dispersal against the population's long axis (i.e., population direction) might affect the respective effective dispersals within the population because the effective dispersals depend on the primary dispersal patterns (reflected as dispersal kernel) and spatial arrangements of suitable habitats (for seed) or conspecific female individuals (for pollen) (Hardy, 2009). If the dispersal is potentially long, the frequency and mean distance of the effective dispersal within such population might increase with the prevailing dispersal direction nearing the population direction and be highest and longest, respectively, when the prevailing dispersal direction is along the population direction. In an opposite way, the frequency and mean distance of the effective dispersals within the population might be the lowest and shortest, respectively, and the immigration rate might be highest when the prevailing dispersal direction is orthogonal to the population direction. Such a process might affect the genetic isolation by distance within the population and thus be reflected as anisotropy of the FSGS. Furthermore, as isotropic FSGS, the anisotropy of the FSGS would be affected by seed and pollen dispersal in different ways and be largely affected by seed dispersal. Riparian forests have important ecological functions (Naiman & Decamps, 1997), and the conservation of the forest has been advocated (Rodewald & Bakermans, 2006). Although past genetic studies on tree species of such forest have indicated useful knowledge for genetic conservation (Goto et al., 2006; Kikuchi et al., 2011; Saeki et al., 2018; Sato et al., 2006), the detailed effects of directionality in separate gene dispersal via seeds and pollen should be taken into account for the clarification of the genetic dynamics and conservation of the genetic diversity.

*Cercidiphyllum japonicum* Sieb. et Zucc. is a dioecious, wind‐pollinated, and wind‐dispersed tree species (Katsuta et al., 1998) that shapes a long and low‐density population along a stream and features riparian forests in Japan together with other tree species (Isagi et al., 2005). This species should be suitable for exploring separate patterns of gene dispersal via seeds and pollen on a large scale. Examined contributions of reproductive individuals even to dispersed offspring can be unambiguously divided into those as seed parent or pollen parent owing to dioecism of the species, which should raise the precision of direct and indirect analyses on gene dispersal. Further, the directionality of gene dispersal via seeds and pollen due to wind direction and their effects on FSGS could be examined using this species. A previous study (Sato et al., 2006) reported the long‐distance gene dispersal of *C*. *japonicum*, with a mean of 129 m and a maximum of 666 m for pollen dispersal and a maximum of 302 m for seed dispersal, as well as a nonsignificant FSGS among adults within the study site. However, the study could not define the accurate pattern of effective seed dispersal due to the seedling sampling sites limited to areas around female trees of *C. japonicum*. Further, the FSGS of adults would reflect the effects of overlapping generations and other demographic dynamics among individuals over a long period, which would make it more difficult to detect the genetic effects of single‐season seed and pollen dispersal. The clarification of a more uncomplicated process, in which the single‐year current gene dispersal affects the consequent FSGS, would require examining dispersed current‐year offspring that have just established.

In this study, we examined patterns of directional seed and pollen dispersal and their separate effects on anisotropy of FSGS among current‐year seedlings of *C. japonicum*. The study used the following steps: (a) examination of a maximum likelihood model to estimate seed and pollen dispersal kernels, taking account of the effects of seed and pollen dispersal directions and sizes of adults using a neighborhood model approach; (b) regression analyses of effective seed and pollen dispersal distances within the population against their respective dispersal directions based on the reconstructed parentage estimated by the maximum likelihood model; (c) isotropic and anisotropic analyses on FSGS among the seedlings; and (d) comparisons between the direction of the weakest FSGS of the seedlings and directions with the most frequent or longest seed dispersal and the most frequent pollen dispersal.

## 2 MATERIALS AND METHODS

### 2.1 Study species

**FIGURE 1:**
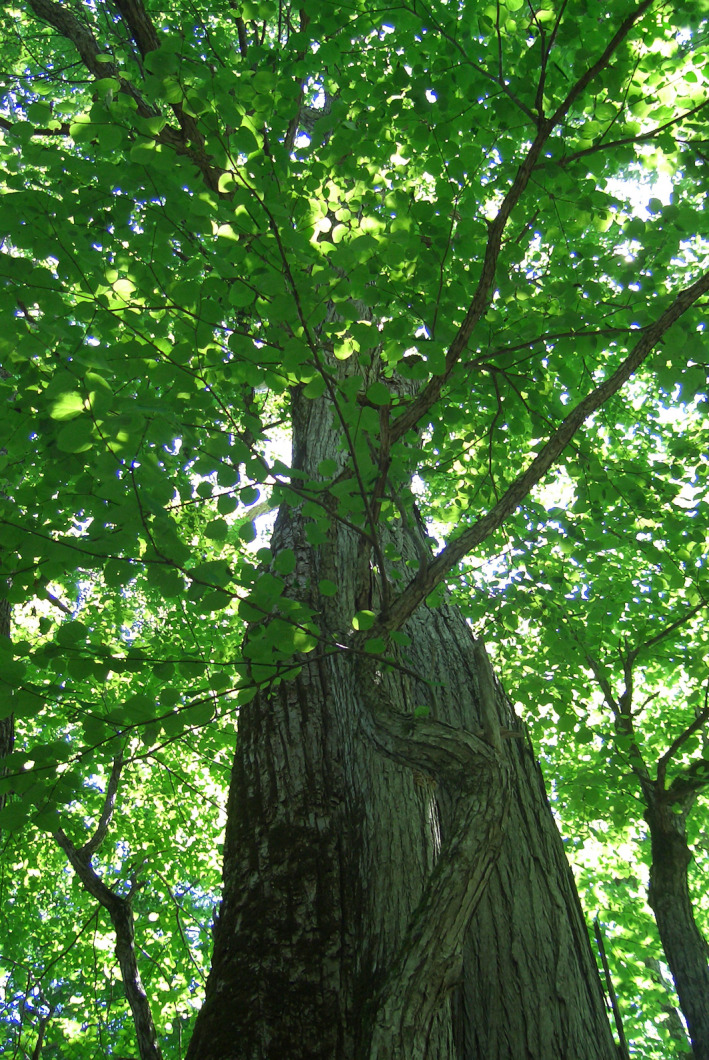
Photograph of *Cercidiphyllum japonicum* tree in the study population

*Cercidiphyllum japonicum* Sieb. et Zucc. (Figure 1, family Cercidiphy‐llaceae) is a deciduous broadleaved tree species that can grow up to 30 m in height and 2 m in diameter (Isagi et al., 2005). The density of adult trees is a few trees per hectare (e.g., 2.5 ha^−1^ in Sato et al., 2006). Its seed has a wing and is about 6 mm long, including the wing, and 2 mm wide (Kubo et al., 2000). The species flowers from April to May, and seeds mature from September (Katsuta et al., 1998). Seed dispersal most frequently occurs in October and November and continues even during winter (Goto, unpublished).

### 2.2 Study site and field methods

**FIGURE 2:**
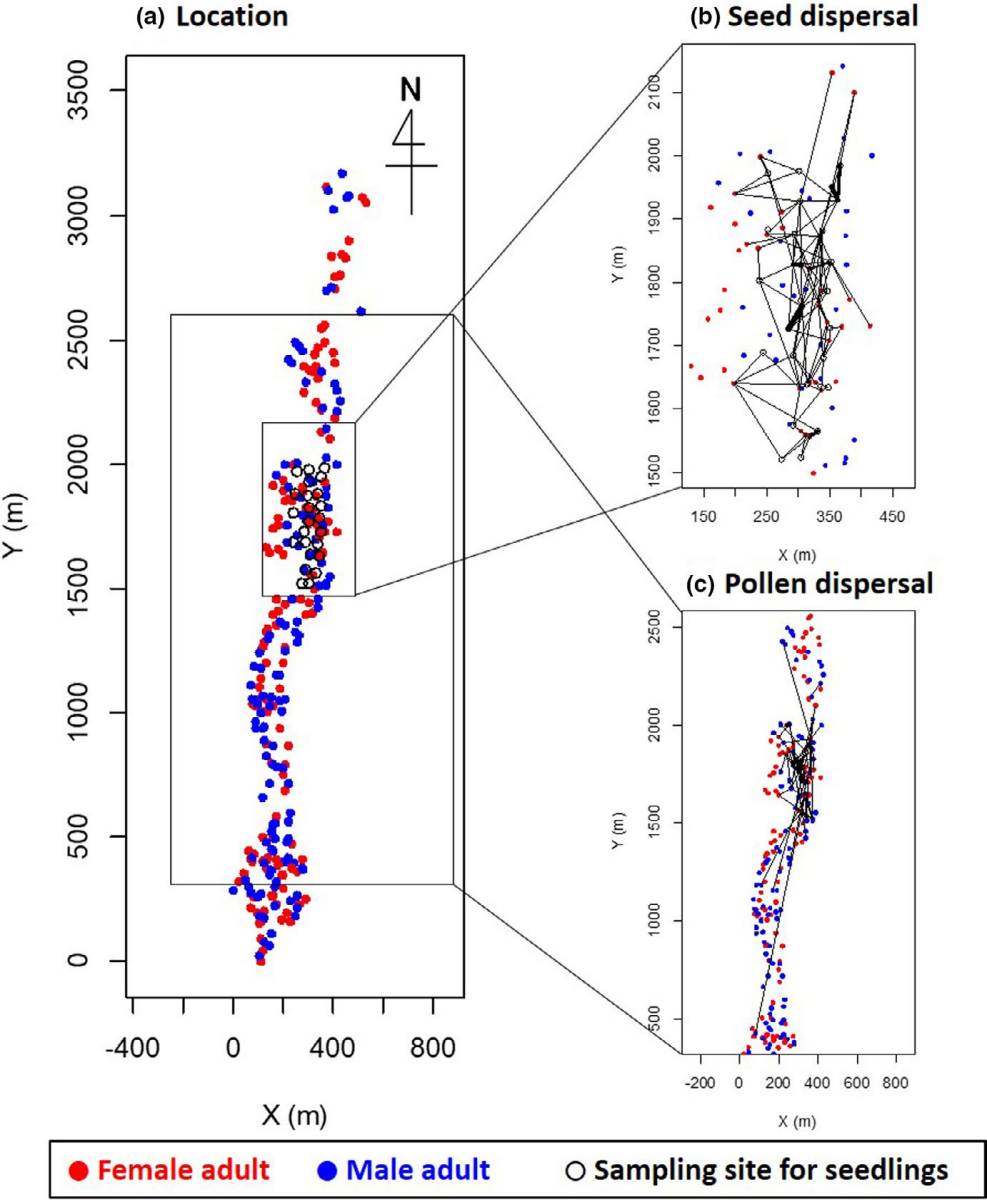
Locations of the female and male adults surveyed, sampling sites for the seedlings, and seed and pollen dispersal estimated by parentage reconstruction with the probability higher than 90%. Red and blue dots indicate the female and male adults, respectively. Black open circles indicate the sampling sites for seedlings. Segments indicate the detected dispersals and the thickness of each segment increases with increasing frequency of dispersal

Our study was conducted in a riparian forest located in Iwanazawa Forest Reserve (43°13′N, 142°34′E), University of Tokyo Hokkaido Forest, in central Hokkaido, Japan. The forest consists of broadleaved trees, such as *C. japonicum, Fraxinus mandshurica, Acer mono, Alnus hirsute*, and *Ulmus davidiana*, and conifers, *Abies sachalinensis* and *Picea jezoensis*. In this forest, genotypes of *C. japonicum* trees within a ca. 10 ha area were already surveyed to study the genetic differentiation among populations throughout the distribution of the species in Japan (Sato et al., 2006). The area was expanded for this study to a width of ca. 200–300 m and a length of ca. 3,200 m (ca. 80 ha) along a stream to encompass the *C*. *japonicum* population. In the expanded area, *C. japonicum* adult trees are spatially aggregated along the stream. We regard this spatial aggregation of *C. japonicum* adult trees as a population. All stems of *C*. *japonicum* with ≥5 cm in diameter at breast height (d.b.h.) within the populations were measured in June 2005. In May 2006, sex expressions of all the individuals in the population with stems having d.b.h. of ≥5 cm were observed by inspecting inflorescences with binoculars, and 131 male and 150 female adult trees were identified and mapped (Figure 2). We sampled leaves from all of the 281 adults in this population. Unsampled *C. japonicum* adult trees outside of and around this population were located more than 100 m away from this population and were scattered at low density. For seedlings, we designed 24 sampling sites in June 2005, the area of which were circular with the radius of about 3 m. The sampling sites were randomly selected from seedling establishment sites, where seedlings could frequently establish, in the middle of the population (Figure 2). The sampling sites were categorized into mounds (8 sites), deer trail (8 sites), fallen logs (3 sites), swamp (3 sites), and others (2 sites). Thirty‐two current‐year seedlings were randomly sampled from each of the 24 sites (768 seedlings in total) in May 2006. The spatial location of each sampling site was regarded as spatial locations of seedlings within the site.

To compare the directions of seed and pollen dispersal to the population direction later, the population direction was calculated as a regression line of model II linear regression (major axis regression) of the y‐coordinate values against the x‐coordinate values of the adult locations under the condition that the direction of the positive y‐axis is the north using the lmodel2 function in lmodel2 package of R version 3.6.3 (R Core Team, 2020). All kinds of estimated directions (i.e., population direction, directions of seed and pollen dispersal, and directions of the FSGS) were expressed by the unit of “2π‐rad” (unit for the ratio of a direction to 2π radian; e.g., 0.5 2π‐rad is the π radian or 180°) clockwise from the north (e.g., 0 2π‐rad is the north and 0.5 2π‐rad is the south) in this study. Consequently, the population direction was 0.016 2π‐rad for the direction from the lower to the upper reaches of the stream, along which the population extends, or 0.516 2π‐rad (0.016 + 0.5 2π‐rad) for the reverse direction (from the upper to lower reaches of the stream).

### 2.3 DNA extraction and microsatellite genotyping

DNA was extracted using a DNeasy Plant Mini Kit (Qiagen, Hilden, Germany) or CTAB method (Murray & Thompson, 1980) with modifications. The genotype of each DNA sample was determined by five microsatellite loci developed for *C*. *japonicum* (MSCJ35, MSCJ86, MSCJ92, MSCJ93, and MSCJ95 [Isagi et al., 2005]). There was no evidence for linkage disequilibrium between loci (Isagi et al., 2005). Polymerase chain reaction (PCR) amplification used the following conditions: initial denaturation at 95°C for 15 min; then 33 cycles of denaturation at 94°C for 30 s, annealing at primer‐specific temperature for 1 min and 30 s, and extension at 72°C for 1 min; final extension at 60°C for 30 min. The sizes of PCR products were determined by capillary electrophoresis using an ABI 3100 Genetic Analyzer and GeneScan software (Applied Biosystems, California, USA). Based on the results of genotyping, the number of individuals that could not be genotyped at each locus (M*_i_*, the number for the *i*th locus), the sum of M*_i_* over the five loci (M*_t_*), and the rate of missing genotypes, which equals to M*_t_* divided by the total number of loci (i.e., 5) multiplied by the total number of examined individuals, were calculated for adults and seedling.

### 2.4 Estimation of genetic diversity for adults and seedlings

Genetic diversity of adults and seedlings across the five loci were estimated using standard population genetic parameters—numbers of different alleles (*A*), observed heterozygosity (*H*_o_), gene diversity (*H*_e_), and inbreeding coefficient (*F*_is_) for each locus and across all loci using Cervus version 3.0.7 (Kalinowski et al., 2007). The frequency of null alleles at each locus for the adult trees was also calculated using Cervus version 3.0.7. Deviation from Hardy–Weinberg equilibrium at each locus for the adults was tested using Genepop ver. 4.2 (Raymond & Rousset, 1995; Rousset, 2008).

### 2.5 Neighborhood model approach for seedlings

Dispersal kernels of seed and pollen dispersal, from which the seedlings originated, were examined simultaneously using a neighborhood model approach based on multilocus genotypes of adults and seedlings (Burczyk et al., 2006) by the NMπ software (Chybicki, 2018). The software implements the following function as dispersal kernel:

$$P\left(r,\theta \right)=f_{R}\left(r\right)f_{\Theta }\left(\theta \right)/r,$$

where *P*(*r*, *θ*) is the probability of dispersal with distance *r* and direction *θ*, *f*_R_(*r*) is the probability of dispersal in radius *r* (in all directions), and *f*_Θ_(*θ*) is the probability of dispersal in direction *θ* (Tufto et al., 1997). The following exponential power function for *f*_R_(*r*) was selected:

$$f_{R}\left(r\right)=rb\exp\left[‐{\left(r/a\right)}^{b}\right]/\left[a^{2}\Gamma \left(2/b\right)\right],$$

where Γ is the gamma function, *a* is the scale parameter, and *b* is the shape parameter that affects the “fatness” of the tail of dispersal distribution (Austerlitz et al., 2004). For *f*_Θ_(*θ*), the software uses von Mises distribution expressed as

$$f_{\Theta }\left(\theta \right)=\exp\left[\kappa \cos\left(\theta ‐\theta_{0}\right)\right]/\left[2\pi I_{0}\left(\kappa \right)\right],$$

where *κ* is the rate parameter, that is, the intensity of directionality in dispersal (Chybicki, 2018), *θ*_0_ is the prevailing direction of dispersal (Chybicki & Burczyk, 2010), and *I*_0_() is the modified Bessel function of the first kind and order zero. Therefore, the exponential‐power‐von Mises function was used as the dispersal kernels. Treating all 281 adults surveyed as neighbors, we estimated maximum likelihood parameters, that is, frequency of immigration (*m*), mean distance (*d*), *b*, *κ*, *θ*_0_, and selection gradient (effect of standardized basal area of an adult on the reproductive success, *g*) for seed and pollen dispersal, and rates of genotyping errors (Chybicki, 2018; Chybicki & Burczyk, 2010). Because *C*. *japonicum* is dioecious, the selfing rate should be 0. The parentage of seedlings was reconstructed by the maximum likelihood model estimated above (Chybicki, 2018). The NMπ software calculates posterior probabilities of most likely parent pairs based on the maximum likelihood model, each of which is either a pair of (a) both candidates within the population, (b) a candidate within the population and an unknown candidate outside the population, or (c) both unknown candidates outside the population. We determined these three types of parent pairs, and a parent or parent pair within the population of each seedling by posterior probabilities higher than 90% based on the maximum likelihood model. To examine the effect of threshold (i.e., the posterior probability for the parent pair of seedlings) on the rates of parentage determination (proportion of categorizing the parent pairs into the above three types of parentages) and estimated rates of seed and pollen immigration from outside of the population, the parentage reconstructions with the other different thresholds of 75%, 50%, and 0% were conducted.

### 2.6 Regression analyses for the distances of effective seed and pollen dispersal within the population against the respective dispersal directions

To examine whether the distances of effective seed and pollen dispersal within the population depend on the respective dispersal directions and estimate the directions with the longest seed and pollen dispersal, the following regression analyses were conducted for seed and pollen dispersal detected within the population by parentage reconstruction with the posterior probability higher than 90%. To conduct more robust examination, the following two models expressed by functions with different shapes were used:

$$D=\beta_{1}\cos\;\left(\theta ‐\beta_{2}\right)+\beta_{3}\;\left(\text{model}\;1\right),$$

$$D=\beta_{4}\exp\left[\beta_{5}\cos\left(\theta ‐\beta_{6}\right)\right]\;\left(\text{model}\;2\right),$$

where *D* is dispersal distance, and *θ* is the dispersal direction. The estimated parameter *β*_4_ must not be lower than 0 because *D* must not be lower than 0. The estimated parameters of *β*_2_ and *β*_6_ are assumed to be equal to or higher than 0 and lower than 2π‐rad. *D* is assumed to be linear and exponential functions of the cosine of the difference between parameter and dispersal directions in models 1 and 2, respectively. When *β*_1_ and *β*_5_ are significantly higher than 0, dispersal direction affects dispersal distance and dispersal distance should be longest at directions of *β*_2_ and *β*_6_, respectively. These analyses were conducted treating directions of explanatory variables and estimated parameters as clockwise directions from the north by nls function of R ver. 3.6.3 (R Core Team, 2020).

To examine whether the detected regression for seed dispersal would be due to the spatial arrangement of the seed parents and sampling locations (i.e., the population shape), the following simulation was conducted. First, *n*_s_ seed dispersals were randomly extracted from 3,600 potential seed dispersals (all the seed dispersals from the 150 females to the 24 sampling sites) with the probability (*P*), calculated by the following function of only distance (*r*) assuming isotropic dispersal:

$$P\left(r\right)=b\exp\left[‐{\left(r/a\right)}^{b}\right]/\left[2\pi a^{2}\Gamma \left(2/b\right)\right],$$

where the parameters were estimated by the neighborhood model approach described previously. *n*_s_ is the number of effective seed dispersals within the population detected by parentage reconstruction with the probability higher than 90%. Second, the mean distance of the simulated seed dispersal was calculated for each of eight dispersal direction classes, from 0.000–0.125 2π‐rad to 0.875–1.000 2π‐rad. Third, the process was repeated 2,000 times, and the actual mean within the population, estimated by the parentage reconstruction, was compared to the simulated means at each direction class.

### 2.7 Isotropic analyses of the FSGS and analysis of the spatial sibling structure

The overall FSGS among adults and seedlings were examined by regressions of kinship coefficient, *F*_ij_ (Loiselle et al., 1995), against the natural logarithm of the spatial distance between individuals (Vekemans & Hardy, 2004). *F*_ij_ values were calculated by SPAGeDi ver. 1.5a (Hardy & Vekemans, 2002). Estimation of regression slopes (*b*_F_, Vekemans & Hardy, 2004) for both adults and seedlings were based on the regression over the whole range of spatial distances among individuals, assuming natural logarithm of the distance between seedlings within sampling sites to be 0. Estimations and significance tests of *b*_F_ used R ver. 3.6.3 (R Core Team, 2020). We calculated *S*p (Vekemans & Hardy, 2004) to evaluate the intensity of FSGS among adults and seedlings using the formula *S*p = *b*_F_/(*F*_1_−1), where *F*_1_ is the mean kinship coefficient between pairs of individuals with distances of 0–50 m. Further, the mean *F*_ij_ for seedlings was calculated for each of the 10 continuous distance classes at 50 m intervals from 0–50 to 450–500 m between sampling sites and within sampling sites (zero distance class). Significances of mean *F*_ij_ values were tested by 1,000 times permutations, in which spatial distances of pairs were permuted randomly among individuals (Hardy & Vekemans, 2002). This analysis for the correlogram of *F*_ij_ was performed using SPAGeDi version 1.5a (Hardy & Vekemans, 2002).

To examine the spatial sibling structure (i.e., spatial aggregations of siblings generated by seed and/or pollen dispersal), the following analyses were conducted based on the seedlings both parents of which could be determined within the population by the parentage reconstruction with the probability higher than 90%. The number​s of pairs for each sibling relationship (i.e., full‐sibling, maternal half‐sibling, paternal half‐sibling, and non‐sibling) at the same distance classes as the *F*_ij_ correlogram were calculated, and the probability of each sibling relationship at each distance class was then calculated by the number of the corresponding sibling pairs divided by the total number of examined pairs at each distance class. The mean *F*_ij_ between the pairs for each sibling relationship was also calculated for the seedlings.

### 2.8 Anisotropic analyses of the FSGS

We examined an anisotropy of FSGS for the adults and seedlings using modified method of Born et al. (2012) based on the bearing analysis method (Falsetti & Sokal, 1993). Born et al. (2012) examined the correlation between kinship coefficient (*F*_ij_) between two individuals and the weighted natural logarithm of spatial distance between individuals (Ln*D*_ij_), but we examined the slope (*b*_F_) of regression of *F*_ij_ against the weighted Ln*D*_ij_, because a decrease in *F*_ij_ with an increase of spatial distance between the individuals (isolation by distance) should characterize the FSGS (Vekemans & Hardy, 2004), not vice versa. The method using regression should assume that the effect of distance between a pair of two individuals on *F*_ij_ is weighted by the similarity between the direction of the pair and the tested direction. The analysis is described below. Natural logarithm of the distance between individuals i and j (Ln*D*_ij_) was transformed by weighting the Ln*D*_ij_ by the squared cosine of the direction *α*_ij_ (Born et al., 2012). *α*_ij_ is the direction between the direction from i to j (*θ*_ij_) and fixed direction (*θ*_k_) (Born et al., 2012; Falsetti & Sokal, 1993), and *θ*_k_ ranges from 0 to 0.45 2π‐rad from the north at intervals of 0.05 2π‐rad in this study. A direction (*θ*) and its reverse direction (*θ* + 0.5 2π‐rad) were treated as the same in this analysis. We assumed Ln*D*_ij_ of seedlings within the sampling sites to be 0 to deal with the absence of *θ*_ij_ of seedlings within sampling sites (i.e., treated *D*_ij_ as 1 m) and treated the weighted Ln*D*_ij_ as 0 excluding the effect of *θ*_ij_. Slope coefficient (*b*_F_) for regression of *F*_ij_ between two individuals against the weighted natural logarithm of spatial distance between the individuals for each *θ*_k_ was tested by R ver. 3.6.3 (R Core Team, 2020) with Bonferroni correction for the number of tests (10). The analyses were conducted for adult pairs between which the distances were shorter than 200 m, and seedling pairs between which the distances were shorter than 100 m, because the maximum distance between pairs varied among directions between the pairs and were limited to about 200 m for adults and about 100 m for seedlings at an approximately east–west direction. Slope coefficients for regressions were plotted against fixed directions (*θ*_k_). We detected the directions of the strongest (*θ*_MAX_) and weakest (*θ*_MIN_) FSGS (Born et al., 2012) by negative lowest and highest slope coefficients, respectively.

## 3 RESULTS

### 3.1 Genetic diversity of adults and seedlings

All 281 adults surveyed and 755 of 768 seedlings sampled were genotyped at five loci with 0.2% and 0.6% of missing genotypes, respectively. The estimated mean ± standard error (*SE*) numbers of different alleles (*A*) over the five loci were 19.0 ± 0.7 for the adults and 20.4 ± 0.5 for the seedlings (Table 1). The estimated mean ± *SE* values of observed heterozygosity (*H*_o_) were 0.830 ± 0.014 for the adults and 0.796 ± 0.022 for the seedlings, and those of gene diversity (*H*_e_) were 0.847 ± 0.014 for the adults and 0.825 ± 0.019 for the seedlings. Inbreeding coefficients (*F*_is_) over all loci for the adults and seedlings were 0.020 and 0.035, respectively. The frequency of null alleles at each locus for the adults ranged from 0.006 to 0.018. Deviations from Hardy–Weinberg equilibrium for the adults were not significant for any loci after Bonferroni correction. Although the number of loci used in this study was somewhat less than other genetic studies, each of the loci showed allelic diversity and low frequency of null alleles, which made these loci adequate for the genetic analyses in this study.

**TABLE 1:** Genetic diversity estimates at each microsatellite locus, and means and standard errors (*SE*) over the five loci among adults and seedlings

Locus	Adult					Seedling				
	*N*	*A*	*H*_o_	*H*_e_	*F*_is_	*N*	*A*	*H*_o_	*H*_e_	*F*_is_
*MSCJ35*	281	21	0.786	0.802	0.020	749	22	0.744	0.769	0.033
*MSCJ86*	279	16	0.832	0.862	0.035	751	19	0.732	0.819	0.106
*MSCJ92*	281	19	0.854	0.867	0.015	753	20	0.833	0.890	0.064
*MSCJ93*	281	20	0.808	0.822	0.017	752	21	0.818	0.796	−0.028
*MSCJ95*	280	19	0.871	0.883	0.014	749	20	0.853	0.851	−0.002
Mean		19.0	0.830	0.847	0.020		20.4	0.796	0.825	0.035
*SE*		0.7	0.014	0.014			0.5	0.022	0.019	

Abbreviations: *A*, number of different alleles; *F*_is_, inbreeding coefficient; *H*_e_, gene diversity; *H*_o_, observed heterozygosity; *N*, number of analyzed individuals.

### 3.2 Potential seed and pollen dispersal estimated by neighborhood model approach

According to the neighborhood model approach using exponential‐power‐von Mises functions as dispersal kernels for both seed and pollen dispersal, the frequency of seed immigration was 0.071 ± 0.013 (*SE*), the mean distance of seed dispersal was 69.1 m, and the prevailing direction of seed dispersal was 0.605 ± 0.023 (*SE*) 2π‐rad (unit for the ratio of a direction to 2π radian, e.g., 0.5 2π‐rad is π radian or 180°, clockwise from the north; Table 2). The frequency of pollen immigration was 0.368 ± 0.023 (*SE*), the mean distance of pollen dispersal was 870.6 m, and the prevailing direction of pollen dispersal was 0.765 ± 0.038 (*SE*) 2π‐rad. The 95% confidence intervals of shape parameters (*b*), rate parameters (*κ*), and selection gradients (*g*) for both seed and pollen dispersal were higher than zero. The estimated rates of genotyping errors at each locus ranged from 0.001 to 0.256. The prevailing direction (*θ*_0_: 0.765 2π‐rad) of potential pollen dispersal and population direction (*Dir*_Pop_: 0.016 or 0.516 2π‐rad) were approximately orthogonal to each other (the difference of 0.25 2π‐rad means π/2 radian or 90 degrees).

**TABLE 2:** Parameters and standard errors (*SE*) for seed and pollen dispersal estimated by the neighborhood model approach using exponential‐power‐von Mises functions

Parameter	Seed dispersal	Pollen dispersal
*m*	0.071 (0.013)	0.368 (0.023)
1/*d*	0.01446 (0.00099)	0.00115 (0.00064)
*d*	69.1	870.6
*b*	0.603 (0.066)	0.256 (0.061)
*κ*	0.513 (0.073)	0.329 (0.115)
*θ*_0_	0.605 (0.023)	0.765 (0.038)
*g*	0.469 (0.042)	0.503 (0.040)

Abbreviations: *b*, shape parameter of dispersal kernel; *d*, mean distance (m); *g*, selection gradient (effect of basal area on reproductive success); *m*, frequency of immigration; *θ*_0_, prevailing direction (dominant angle of von Misses distribution) clockwise from the north of dispersal (2π‐rad, unit for the ratio of a direction to 2π radian); *κ*, intensity rate of directionality in dispersal (rate parameter of von Misses distribution).

### 3.3 The parentage of seedlings and effective seed and pollen dispersal reconstructed by the maximum likelihood model

As the result of parentage reconstruction for 755 seedlings based on the maximum likelihood model, the three types of parent pairs (see Section 2) for 306 seedlings were determined with posterior probabilities higher than 90% (Table 3). The parent pairs of the remaining 449 seedings could not be categorized into the three types of parentage with the threshold. In other words, whether each of the parents was located within or outside the population could not be determined for the 449 seedlings with the threshold. These 449 seedings were discarded in the subsequent analyses on effective dispersals within the population to increase the accuracy of the analyses, and only the data of the remaining seedlings were used for the analyses. Of the remaining 306 seedlings with the probability higher than 90%, 17 (5.6%) were estimated to have both parents outside and 289 (94.4%) were estimated to have at least a seed parent within the population. Thus, the estimated seed immigration rate was 5.6%. Of the 289 seedlings, 89 (30.8%) were estimated to have pollen parents outside and 200 (69.2%) were estimated to have pollen parents within the population. Thus, the estimated pollen immigration rate was 30.8%. Twenty‐seven female adult trees were estimated to have contributed to the 289 seedlings as seed parents, and 30 male adult trees were estimated to have contributed to the 200 seedlings as pollen parents (Figure 2). The mean ± standard deviation (*SD*) distance of seed dispersals within the population calculated for the 289 seedlings was 50.9 ± 37.4 m and that of pollen dispersal calculated for the 200 seedlings was 86.3 ± 118.4 m. The longest distance of seed dispersal within the population was 250.9 m, whereas that of pollen dispersal was 1,234.7 m.

**TABLE 3:** Numbers of seedlings categorized into three types of parentages and seed and pollen immigration rates, estimated by parentage reconstructions with the different thresholds of probabilities

Threshold	90%	75%	50%	0%
Categorized	306 (40.5%)	434 (57.5%)	621 (82.3%)	755 (100.0%)
Having at least a seed parent within the population	289	404	578	705
^1^Having a parent pair within the population	200	263	369	451
^2^Having a pollen parent outside the population	89	141	209	254
^3^Having a parent pair outside the population	17	30	43	50
Not categorized	449 (59.5%)	321 (42.5%)	134 (17.7%)	0 (0.0%)
Rate of immigration from outside of the population				
^4^Seed dispersal	5.6%	6.9%	6.9%	6.6%
^5^Pollen dispersal	30.8%	34.9%	36.2%	36.0%

Note

^1, 2, and 3^The three types of parent pairs.

^4^The number of seedlings having a parent pair outside the population divided by the number of categorized seedlings.

^5^The number of seedlings having a seed parent within the population but a pollen parent outside divided by the number of seedlings having at least a seed parent within the population. The proportions of seedlings having categorized or noncategorized parent pairs to the total of 755 seedlings analyzed were indicated in parentheses.

As a result of parentage reconstructions with different thresholds of probabilities (90%, 75%, 50%, and 0%; Table 3), the proportion of seedlings (40.5%–100%) the parent pair types of which could be determined increased with decreasing probability of the threshold. Although the rate of indetermination of parent pair types depended on the threshold, the rates of seed immigration (5.6%–6.9%) and pollen immigration (30.8%–36.2%) estimated by parentage reconstruction were similar among the different thresholds and near the respective rates estimated by the neighborhood model approach.

### 3.4 Effects of dispersal directions on the distances of effective seed and pollen dispersal within the population

**FIGURE 3:**
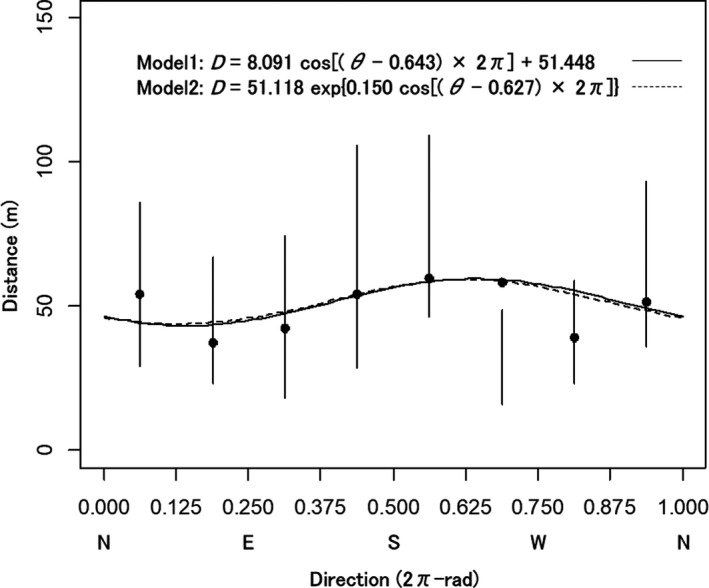
Mean distances of seed dispersal within the population estimated by parentage reconstruction with the probability higher than 90% and the 2,000 times simulations with the isotropic dispersal kernel at direction classes. The direction is clockwise from the north and indicated by the unit of 2π‐rad (unit for the ratio of a direction to 2π radian; e.g., 0.5 2π‐rad is π radian or 180°). At each direction class, a black circle indicates the mean estimated by parentage reconstruction and a vertical bar indicates the entire range of 2,000 simulated means. The solid curve represents the dispersal distance (*D*) as the linear function of the cosine of the difference between the parameter direction and dispersal direction (*θ*) in model 1 fitted to the data estimated by the parentage reconstruction. The dashed curve represents the *D* as the exponential function of the cosine of the difference between the parameter direction and *θ* in model 2. N, E, S, and W below the horizontal coordinate values indicate the north, east, south, and west directions, respectively, corresponding to the positions of the coordinate values

According to regressions of effective seed dispersal distance against the dispersal direction by two models based on the reconstructed parentage of the 289 seedlings, of which seed parents could be determined within the population with the probability higher than 90%, the effects of the direction were detected in both models [*p* < .05 for *β*_1_ (*t* = 2.583) and *β*_5_ (*t* = 2.519); Figure 3], and the dispersal distance should be longest at the direction of *β*_2_ [0.643 ± 0.065 (*SE*) 2π‐rad clockwise from the north] in model 1 and *β*_6_ [0.627 ± 0.071 (*SE*) 2π‐rad] in model 2. According to regressions of effective pollen dispersal distance against the dispersal direction by the two models based on the reconstructed parentage of the 200 seedlings, of which both parents could be determined within the population, any significant effect of the direction was not detected.

According to the simulation (578,000 extracted seed dispersals; i.e., extracting 289 seed dispersals at 2,000 times) for effective seed dispersal within the population, the actual mean distance (58.4 m) of the seed dispersals was significantly longer than those of the simulated dispersals (16.3–48.6 m) at the 0.625–0.750 2π‐rad direction class (*p* < .001). The direction class was consistent with the directions with the longest dispersal estimated by the abovementioned regressions of model 1 (0.643 2π‐rad) and 2 (0.627 2π‐rad).

### 3.5 Overall trends in the FSGS and spatial sibling structure

**FIGURE 4:**
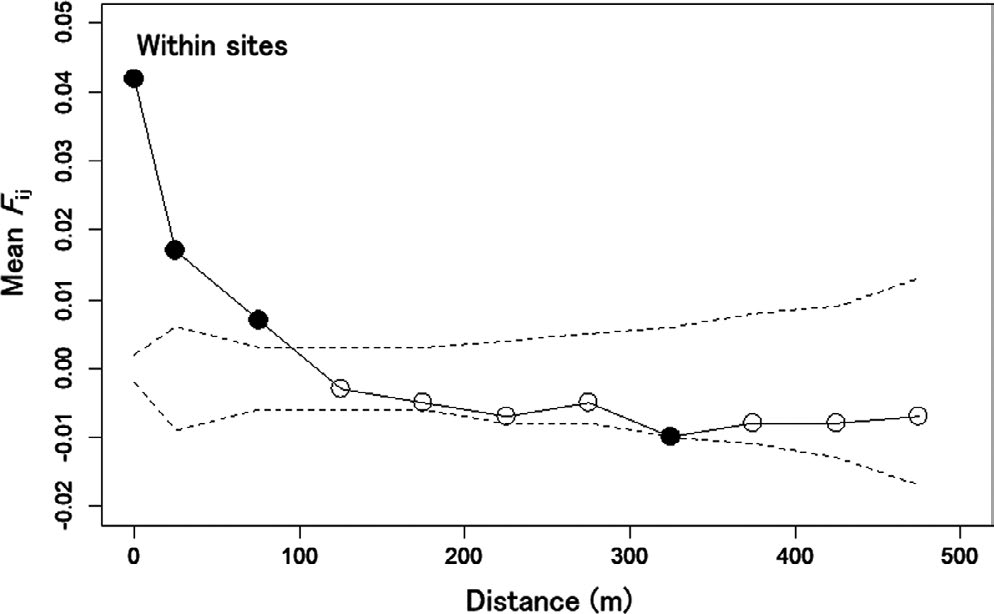
Mean *F*_ij_ values between pairwise seedlings against spatial distances between the pairs. The mean value at distance class zero corresponds to the mean value of the pairs within sampling sites. The dashed lines indicate the 95% confidence intervals generated by 1,000 times permutations. Filled and unfilled circles indicate the significant and nonsignificant values at a probability of .05, respectively

Isotropic analyses of FSGS among all 281 adults and 755 seedlings showed significantly negative *b*_F_ values, slopes for regressions of kinship coefficients (*F*_ij_) against natural logarithm of the spatial distances between individuals, for both adults (*b*_F_ = −0.011, *t* = −23.88, *p* < .001) and seedlings (*b*_F_ = −0.009, *t* = −58.19, *p* < .001). The *Sp* of adults and seedlings were 0.012 and 0.009, respectively. The *F*_ij_ correlogram of seedlings showed significantly high mean *F*_ij_ values within sampling sites and for 0–50 to 50–100 m distance classes between sampling sites (permutation test, *p* < .05; Figure 4), and the value within the sites was highest (0.042). The *F*_ij_ value decreased as distance increased and was significantly low for the 300–350 m distance class.

**FIGURE 5:**
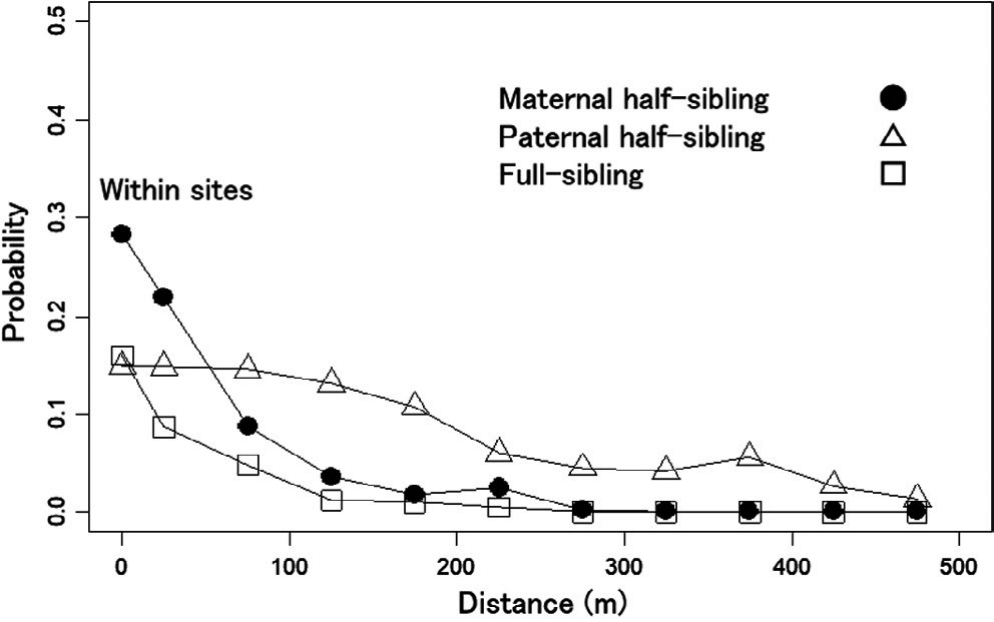
Probabilities of full‐sibling, maternal half‐sibling, and paternal half‐sibling between seedling pairs against the spatial distance between the pairs among 200 seedlings, of which both parents could be determined within the population by parentage reconstruction with the probability higher than 90%. The probabilities at distance class zero correspond to the probabilities of the pairs within sampling sites. Filled circle, unfilled triangle, and unfilled square represent the probabilities of maternal half‐sibling, paternal half‐sibling, and full‐sibling, respectively

As the result of sibling analyses based on the 200 seedlings (19,900 pairs), of which both parents could be determined within the population by the parentage reconstruction with the probability higher than 90%, the probabilities between the seedling pairs of full‐sibling, maternal half‐sibling, and paternal half‐sibling were highest within the sampling sites (0.160, 0.283, and 0.149, respectively; Figure 5). These probabilities between sampling sites decreased as the distance class increased. Although the probabilities of full‐sibling and maternal half‐sibling decreased more rapidly and reached zero at distance classes of 250–300 and 350–400 m, respectively, that of paternal half‐sibling decreased more gradually and higher than zero (0.013) even at the longest distance class (450–500 m). The mean ± *SE* of *F*_ij_ values between pairs were 0.219 ± 0.006 for full‐sibling, 0.114 ± 0.003 for maternal half‐sibling, 0.098 ± 0.002 for paternal half‐sibling, and −0.010 ± 0.001 for non‐sibling.

### 3.6 Anisotropy of the FSGS

**FIGURE 6:**
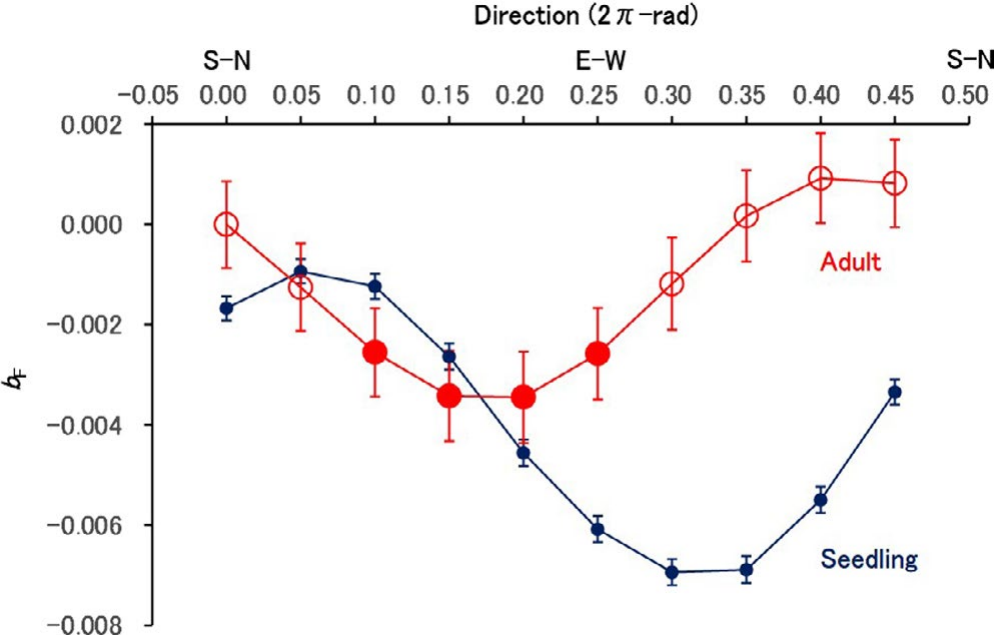
Slope coefficients for regressions of kinship coefficients of adult pairs and seedling pairs against the weighted natural logarithm of spatial distances between the pairs plotted against the tested directions. The adult pairs and seedling pairs with spatial distances shorter than 200 and 100 m, respectively, were chosen for the calculations. The estimated values for adults and seedlings are shown as red and blue circles, respectively. Filled and unfilled circles indicate the significant and nonsignificant values at a probability of .05 after Bonferroni correction, respectively. Error bars indicate standard errors. The tested direction is clockwise from the north and indicated by the unit of 2π‐rad (unit for the ratio of a direction to 2π radian; e.g., 0.5 2π‐rad is π radian or 180°). The tested direction ranged from 0 to 0.45 2π‐rad, and direction from i to j (*θ*) and the reverse direction (direction from j to i, *θ* + 0.5) were treated as the same in this analysis. S‐N and E‐W above the horizontal coordinate values indicate dual directions of south‐north and east‐west, respectively, corresponding to positions of the coordinate values

As a result of anisotropic analysis of FSGS among seedlings, slopes (*b*_F_) for regressions of kinship coefficient (*F*_ij_) values against weighted natural logarithms of spatial distances between individuals varied with tested directions (Figure 6). The *b*_F_ values for the seedlings were significantly negative (*p* < .01 after Bonferroni correction) at all tested directions (*θ*_k_ = 0.00–0.45 2π‐rad clockwise from the north) and lowest at the direction of 0.3 2π‐rad (*θ*_MAX_: the direction at which *b*_F_ was lowest). The *b*_F_ value was negative but highest in the direction of 0.05 2π‐rad (*θ*_MIN_: the direction at which *b*_F_ was highest), which was orthogonal to *θ*_MAX_ (The difference of 0.25 2π‐rad means π/2 radian or 90 degrees). Because a direction (e.g., *θ* 2π‐rad) and its reverse direction ([*θ* + 0.5] 2π‐rad) were treated as the same in this analysis, FSGS among seedlings was strongest at the direction (*θ*_MAX_) of 0.3 or 0.8 2π‐rad, and weakest at the direction (*θ*_MIN_) of 0.05 or 0.55 2π‐rad. In contrast, the *b*_F_ values for the adult trees were significantly negative (*p* < .05 after Bonferroni correction) only at the four directions (*θ*_k_ = 0.10–0.25 2π‐rad) and lowest at the direction (*θ*_MAX_) of 0.20 2π‐rad. The lowest *b*_F_ (i.e., *b*_F_ at *θ*_MAX_) was much less negative (much higher) for adults (−0.003 ± 0.001 *SE*) than for seedlings (−0.007 ± 0.000 *SE*).

## 4 DISCUSSION

### 4.1 Patterns of potential seed and pollen dispersal

The results from the neighborhood model approach indicated that the frequencies of potential seed and pollen dispersal increased with the dispersal directions nearing the respective prevailing directions (direction with the most frequent dispersal) and that a female or male adult having a larger size was more likely to be seed parent or pollen parent of seedlings, respectively. Results from the neighborhood model approach also indicated a mean distance of potential pollen dispersal (870.6 m), over ten times longer than that of seed dispersal (69.1 m), and the frequency (36.8%) of immigration from outside of the neighborhood (the surveyed population) for pollen dispersal, over five times higher than that (7.1%) for seed dispersal. These results would indicate that potential seed dispersal was much more spatially limited compared to pollen dispersal.

We compared mean distances of seed and pollen dispersal by the wind of *C*. *japonicum* in this study with those of other studies on forest tree species using dispersal kernels. Mean distance of seed dispersal in this study (69.1 m) is longer than species with primarily gravity‐dispersed seeds (18.0 m [Nakanishi et al., 2015]) and is in reported range of wind‐dispersed species (31–127 m [Guidugli et al., 2016; Kitamura et al., 2018]), but shorter than that with animal‐dispersed seeds (98–135 m [Browne et al., 2018; Oddou‐Muratorio & Klein, 2008]). Mean distance of pollen dispersal in this study (870.6 m) is much longer than other wind‐pollinated species (60–417 m [Inanaga et al., 2014; Kassa et al., 2018; Kitamura et al., 2018]) and is in reported range of animal‐pollinated species with mobile‐pollinators (227–1487 m [Breed et al., 2015; Browne et al., 2018; Guidugli et al., 2016; Nakanishi et al., 2020]). Therefore, although the distance of the potential seed dispersal of *C. japonicum* would be moderate among wind‐dispersed tree species, that of potential pollen dispersal would be extensive among wind‐pollinated tree species.

### 4.2 Overall trends in the FSGS and spatial sibling structure

There was a significant FSGS among *C. japonicum* adults in this population. The *Sp* (0.012) for adults was approximately intermediate in the reported range (0.0102 ± 0.0096 *SD*) of tree species (Vekemans & Hardy, 2004), but relatively high in wind‐pollinated and wind‐dispersed tree species (0.00196–0.01076 [Vekemans & Hardy, 2004]; 0.00332 [Lian et al., 2008]; and 0.001 [Kitamura et al., 2018]). The intensity of FSGS decreases with both increasing gene dispersal distance and reproductive individual density, through an increasing degree of overlap between individual “gene shadows” (Oddou‐Muratorio & Klein, 2008). Therefore, although wind seed dispersal and wind pollination may induce long‐distance gene dispersal that weakens FSGS, low density of parents that produce examined individuals might strengthen FSGS. A previous study by Sato et al. (2006) showed nonsignificant FSGS among *C*. *japonicum* adults within the other population. Although the difference of FSGS between studies of *C*. *japonicum* might be due to differences of statistical approaches, numbers of examined individuals, or examined scales, lower density of the previous generation in this population might induce the significant and relatively strong FSGS. Further, Born et al. (2012) suggested variation of dispersal distance within species, especially with wind dispersal. Thus, the distance of seed and/or pollen dispersal in this study population might be shorter than the study by Sato et al. (2006) due to the wind pattern in this population. There was also a significant FSGS among the current‐year seedlings. The *Sp* (0.009) of seedlings in this study was in the reported range for seedlings (current and 2‐year‐old) of other wind‐pollinated and wind‐dispersed tree species (0.00435 [Lian et al., 2008]; and 0.012 [Kitamura et al., 2018]).

Because the mean *F*_ij_ between seedling pairs of maternal half‐sibling, paternal half‐sibling, and full‐sibling were much higher than non‐sibling, the spatial aggregation of such sibling can heighten the mean *F*_ij_ at short distance classes. However, the rapid decrease pattern of *F*_ij_ against the distance for seedlings (*F*_ij_ correlogram) was much more similar to probabilities of the full‐sibling and maternal half‐sibling than the paternal half‐sibling. Therefore, the decrease pattern, which would indicate the existence and intensity of FSGS, would reflect the aggregations of maternal siblings, including full siblings around seed parents. The significances of the *F*_ij_ values only at short distance classes (0–100 m) in the correlogram would also reflect the aggregations of maternal siblings, considering the mean distance of potential seed (69.1 m) and pollen dispersal (870.6 m). However, because the distance‐dependent pollen dispersal would produce full‐siblings within the maternal siblings aggregated around seed parents and consequently heighten the *F*_ij_ within sampling sites and between sampling sites at short distance classes, pollen dispersal would also strengthen the FSGS. Such a process has been indicated by another study (Nakanishi et al., 2009). Therefore, seed dispersal critically affects shaping the FSGS as in other studies (Browne et al., 2018; Grivet et al., 2009; Nakanishi et al., 2009). This result is expected because seed dispersal transports both male and female gametes and determines the final locations of genotypes (Browne et al., 2018; Grivet et al., 2009). Pollen dispersal may frequently exceed the examined scale for seedlings, as indicated by the mean distance (870.6 m).

### 4.3 Directionalities of seed and pollen dispersal and their separate effects on anisotropy of FSGS

**FIGURE 7:**
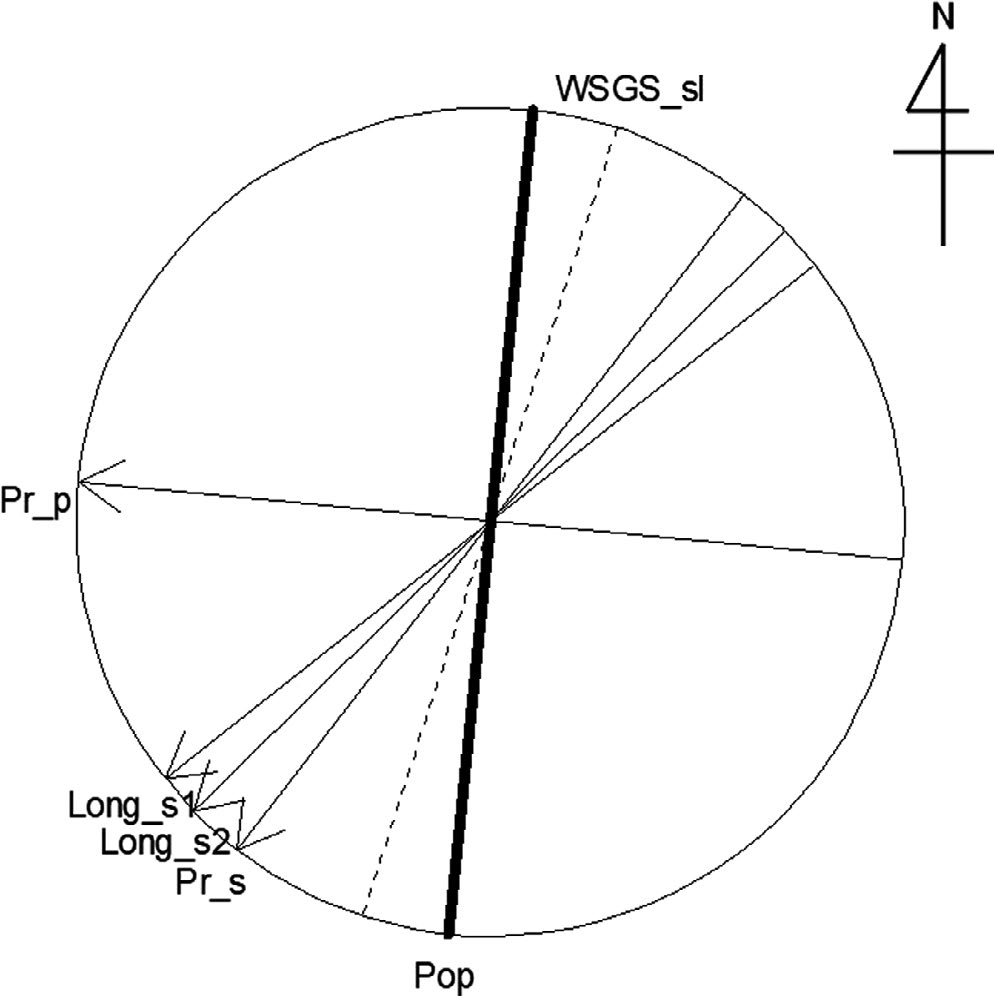
Directions of the population and the weakest spatial genetic structure among the seedlings, prevailing directions of the potential seed and pollen dispersal, and directions of the longest seed dispersal within the population estimated by the two models. The directions of the population (Pop) and the weakest spatial genetic structure among the seedlings (WSGS_sl) are indicated by thick solid and dashed lines, respectively. Prevailing directions of the potential pollen (Pr_p) and seed dispersal (Pr_s), and directions of the longest seed dispersal within the population estimated by the model 1 (Long_s1) and 2 (Long_s2), are indicated by arrows

The frequencies of potential seed and pollen dispersal were estimated to increase with the dispersal directions nearing the respective prevailing directions (direction of the most frequent dispersal). High wind speed and/or long persistence time at particular directions would heighten frequencies of physical seed (Bullock & Clarke, 2000) and pollen dispersal (Damialis et al., 2005; Silva Palacios et al., 2000) via wind. Thus, estimated prevailing directions of potential seed and pollen dispersal in this population may be approximately consistent with wind directions with the highest wind speed and/or longest persistence time, that is, the most effective wind directions, during their respective dispersals. Further, the distance of effective seed dispersal within the population was estimated to depend on dispersal direction and be longest near the prevailing direction of seed dispersal (Figure 7). The simulation analysis for seed dispersal also suggested the effect of the directionality in seed dispersal on the dispersal distance rather than the effect of the spatial arrangement of seed parents and sampling sites for seedlings (i.e., effect of population shape or direction). Effective seed dispersal would occur over longer distances near the most effective wind direction. However, the difference of the prevailing directions between seed and pollen dispersal might indicate the temporal fluctuation of the wind direction between the periods of seed dispersal (most frequently occurs in October and November and continues even during winter) and pollen dispersal (from April to May). Furthermore, although prevailing wind would induce long‐distance gene dispersal (Born et al., 2012), turbulence may affect wind direction (Born et al., 2012) and induce long‐distance seed dispersal (Bullock & Clarke, 2000). However, unfortunately, neither the prevailing wind direction nor turbulence during the seed and pollen dispersal, from which the examined seedlings originated, at the population has been measured.

The relationship between the population direction and the dispersal direction should affect the effective seed and pollen dispersal in the long and relatively narrow studied population. The studied population was comparatively long (ca. 3,200 m) to cover the dispersal along the population direction but much narrower (ca. 200–300 m) to cover the dispersal orthogonal to the population direction, and the pollen dispersal was potentially long (mean = 870.6 m), but the prevailing direction of the dispersal was almost orthogonal to the population direction (Figure 7). Such condition may mostly reduce the frequency and mean distance within the population and increase the immigration rate (36.8%). The mean distance of all effective pollen dispersals, including immigration, which may frequently occur in the prevailing direction, should be much longer than that estimated within the population. In contrast, because the potential seed dispersal was spatially limited and the population direction was nearer the prevailing direction of seed dispersal than that of pollen dispersal, the surveyed range could cover a large part of effective seed dispersal at any directions, as indicated by low frequent seed immigration (7.1%), although there might be a possibility that the low frequent but long‐distance seed immigration occur.

The FSGS among the seedlings within 100 m varied with directions between individuals and was strongest at the *θ*_MAX_ and weakest at the *θ*_MIN_, which is orthogonal to the *θ*_MAX_. Born et al. (2012) showed a direction (*θ*_MIN_) of the weakest FSGS perfectly aligned with the prevailing wind direction, at which gene dispersal may occur over a long distance, at a site. In this study, the directions of the most frequent and longest seed dispersal were near each other and to the *θ*_MIN_ of seedlings, although the prevailing direction of pollen dispersal was more different from the *θ*_MIN_ (Figure 7). These results suggest that high‐frequency and long‐distance seed dispersal around the prevailing direction weakens the FSGS near the direction by increasing the overlaps of seed shadows. Therefore, directional seed dispersal would mainly affect the direction of FSGS among the seedlings. Although extensive pollen dispersal might weaken FSGS (Ueno et al., 2000), such effect of directional pollen dispersal within a population might depend on the prevailing dispersal direction against the population direction in a long and narrow population. In this study, although the pollen dispersal was potentially long, the prevailing dispersal direction orthogonal to the population direction might have caused restricted distance of effective pollen dispersal within the population and thus limited the consequent effect of weakening FSGS. This process might partly explain the difference between the prevailing direction of pollen dispersal and *θ*_MIN_ of the seedlings.

However, the anisotropy of the FSGS was much weaker for the adults than seedlings, probably because the examined adults established more extensively (over all the population) and during a much longer period than the seedlings. The spatial and/or yearly variations in the prevailing directions of the dispersals which generated the adults might weaken the anisotropy of FSGS among adults, and the prevailing direction of the dispersals estimated for the seedlings might be local and/or yearly fluctuate.

## 5 CONCLUSIONS

The potential seed and pollen dispersal estimated in *C*. *japonicum* occurred over shorter and much longer distances, respectively, but estimated frequencies of both dispersals and estimated distance of the effective seed dispersal within the population depended on dispersal directions, probably due to the effective wind directions. Further, our results suggest that spatially limited seed dispersal should generate significant FSGS among the seedlings by the aggregation of maternal siblings, but long‐distance and frequent seed dispersal at around the prevailing direction of dispersal weakens the FSGS at near the direction, although such effect of pollen dispersal could not be detected. Therefore, spatially limited but directional seed dispersal would determine the existence and direction of FSGS among the seedlings. Extensive pollen dispersal might weaken FSGS, thus it might prevent local genetic differentiation within populations. However, in this study, although the pollen dispersal was potentially long, the prevailing dispersal direction orthogonal to the population direction might have caused restricted distance of effective pollen dispersal within the population and thus limited the consequent effect of weakening FSGS. In this way, dispersal directionality might affect distance of pollination within a population and the consequent genetic effect in a long but narrow population of wind‐pollinated tree species in riparian forests. Therefore, the directionality of pollen dispersal should be taken into account for studies on the genetic dynamics and genetic conservation of such populations.

## CONFLICT OF INTEREST

There were no conflicts of interest.

## AUTHOR CONTRIBUTIONS

**Atsushi Nakanishi:** Conceptualization (equal); Formal analysis (lead); Methodology (lead); Writing‐original draft (lead); Writing‐review & editing (lead). **Susumu Goto:** Conceptualization (equal); Data curation (equal); Investigation (equal); Project administration (lead); Resources (lead); Supervision (lead); Writing‐original draft (equal); Writing‐review & editing (equal). **Chikako Sumiyoshi:** Data curation (lead); Investigation (lead); Resources (lead); Writing‐original draft (equal); Writing‐review & editing (equal). **Yuji Isagi:** Project administration (lead); Supervision (lead); Writing‐original draft (equal); Writing‐review & editing (equal).

## Data Availability

Field data and microsatellite genotypes are available from the Dryad Digital Repository (https://doi.org/10.5061/dryad.zcrjdfnb7).
